# You are invited to submit…

**DOI:** 10.1186/s12916-015-0423-3

**Published:** 2015-08-04

**Authors:** David Moher, Anubhav Srivastava

**Affiliations:** Clinical Epidemiology Program, Ottawa Hospital Research Institute, Ottawa, Canada; School of Epidemiology, Public Health and Preventive Medicine, University of Ottawa, Ottawa, Canada; University of Toronto, Toronto, Canada; Clinical Epidemiology Program, Ottawa Hospital Research Institute, The Ottawa Hospital, General Campus, 501 Smyth Rd, Room L1288, Ottawa, ON K1H 8L6 Canada

**Keywords:** Academic incentives and rewards, Dissemination of research, Invitations, Potential predatory journals

## Abstract

The academic community is under great pressure to publish. This pressure is compounded by high rejection rates at many journals. A more recent trend is for some journals to send invitations directly to researchers inviting them to submit a manuscript to their journals. Many researchers find these invitations annoying and unsure how best to respond to them. We collected electronic invitations to submit a manuscript to a journal between April 1, 2014, and March 31, 2015. We analyzed their content and cross-tabulated them against journals listed in Beall’s list of potential predatory journals. During this time period, 311 invitations were received for 204 journals, the majority of which were in Beall’s list (n = 244; 79 %). The invitations came throughout the calendar year and some journals sent up to six invitations. The majority of journals claimed to provide peer review (n = 179; 57.6 %) although no mention was made of expedited review process. Similarly, more than half of the journals claimed to be open access (n = 186; 59.8 %). The majority of invitations included an unsubscribe link (n = 187; 60.1 %). About half of the invitations came from biomedical journals (n = 179). We discuss strategies researchers and institutions can consider to reduce the number of invitations received and strategies to handle those invitations that make it to the recipients’ inbox, thus helping to maintain the credibility and reputation of researchers and institutions.

## Background

There are a multitude of reasons why academic authors want to publish their research. A basic motive is to share knowledge with colleagues and others. Another reason might include wanting the research to influence healthcare practice and/or policy. Finally, an unfortunate and misguided, yet very real, incentive is to withstand the current ‘publish or perish’ mantra. The number of publications, regardless of the completeness and transparency of the report [1] or whether the methods can be replicated [2, 3], is a strong motivator, often used to help assess promotion and tenure.

Barriers to publication include knowing which of a large number of potential journals might be interested in the content being reported and very high rejection rates, particularly in luxury journals [4]. Some journals are trying to reduce these barriers by actively courting prospective authors. For example, a journal might send an electronic invitation to their distribution list soliciting manuscripts for a specific thematic issue. The amount of invitations is growing rapidly; researchers are being inundated with electronic invitations from journals or publishers requesting them to submit manuscripts. Often, the invitations include a flattering personalized greeting and information about the prestige of the invitee. The invitations can be annoying, requiring time to read and deciding whether or not to act on the offer. Furthermore, it is unclear as to whether the manuscripts published by these journals add value to either the journals or the submitting authors. Authors may be early in their research career and naively think publishing in these journals will help disseminate their research findings. Fortunately, these journals are rarely indexed by legitimate databases [5] such as PubMed.

Despite its widespread nature, little has been published to date about this phenomenon, including whether the rise in these invitations is associated with the rise of predatory journals (journals exploiting gold open access, providing no or non-sensible peer review, undercutting standard article processing charges, while frequently publishing rubbish [6, 7]), or how prospective authors and their institutions should deal with them.

To better understand the characteristics of these invitations, we collected those received by one of the authors (DM) over a 1-year period.

## Methods

We did not have an *a priori* written protocol for this project. Between April 1, 2014, and March 31, 2015, we sequentially collected (electronically) all invitation letters to submit a manuscript to a journal. During the data collection period, we developed and refined a data collection form listing the date and frequency of the invitation categorized by month, the name of the journal and associated publisher, whether the journal and/or publisher was indexed on Beall’s list of suspected predatory journals [8], any special salutation associated with the invitation, whether the invitations were from a biomedical journal (assessed by reading each invitation for any mention of biology or medicine) and, if so, the broad ICD-10 categorization, a claim of being open access, whether peer review was mentioned and, if so, whether there was mention of an expedited review process, and the existence of an unsubscribe link. Once the data collection period ended, the invitations were exported to Microsoft Excel, the data cleaned, and descriptive analyses completed. Beall’s list was used (during the week of April 13, 2015) to cross reference invitations with predatory journals and publishers.

## Results

**Fig. 1:**
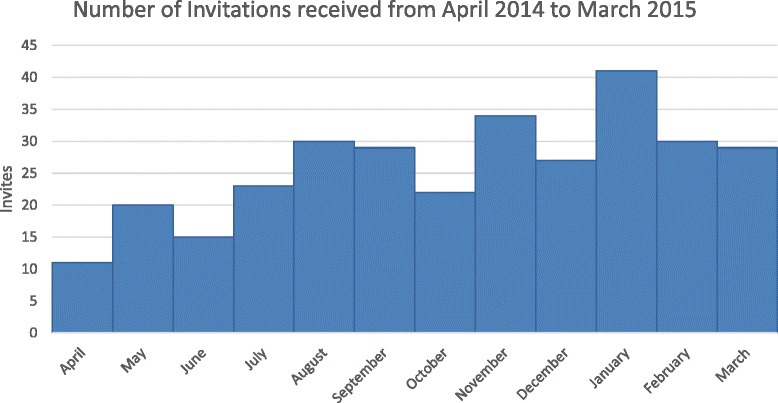
Histogram of invitations by month

During the 12-month period, 311 invitations were received. Two invitations had the correct e-mail address but were addressed to a different person. Invitations were received from 204 unique journals, 57 of which accounted for half of all the invitations (the number of invitations received from each journal ranged from 1 to 6). The majority of invitations (n = 244; 78.5 %) were from journals on Beall’s list. The remaining invitations were from journals that shared many similar characteristics to the known predatory ones, including but not limited to flattering salutations, claims that they had read the recipient’s papers despite being out of the journal’s claimed area of study, awkward sentence structure and spelling mistakes, and extremely general topics. The invitations were received throughout the year peaking in January 2015 (Fig. 1). The 17 most prevalent publishers sending invitations were included in Beall’s list. The 13 most frequent publishers accounted for the majority of invitations (n = 172; 55.3 %; Table 1) and 106 invitations started with special greetings, such as “eminent”, “prominent”, or “expert”, or made reference to the recipient’s “valuable publications”. The majority of invitations claimed the journal provided peer review (n = 179; 57.6 %) although none mentioned anything about an expedited review process. More than half of the invitations claimed to be from open access journals (n = 186; 59.8 %). The majority of invitations (n = 187; 60.1 %) included an unsubscribe link.

**Table 1:** 13 publishers sending the most frequent invitations

Publisher	Frequency
OMICS Publishing Group	69
SciDoc Publishers	21
Jacobs Publishing	13
MedCrave	10
Center for Promoting Ideas	9
Aperito Online Publishing	7
Austin Publishing Group	7
Enliven Archive	6
Journals Club	6
Ommega Publishers	6
Openventio Publishers	6
Science and Education Publishing	6
Symbiosis Online Publishing	6

## Discussion

Within the category of biomedical journals (n = 179), the most common invitations that were easily classifiable pertained to endocrine disorders and metabolic disorders (n = 21; 11.8 %). However, 78 (43.6 %) invitations could not be easily categorized, spanning multiple, if not all, categories of the ICD-10 Electronic invitations to submit a manuscript to a journal are a common, and potentially irritating, occurrence. One of us received more than one invitation daily during the work week, the vast majority coming from predatory journals, most of which claimed to be open access and to provide peer review. About a fifth of the invitations were not on Beall’s list. It is likely that these journals are simply not targeting enough individuals to be discovered and placed on the list yet, or have just started publishing recently (as many of these invitations claimed that the submitted manuscript would be published in the inaugural issue of the suspected predatory journal). There is a substantial growth in new suspected predatory journals, particularly in certain parts of Asia [9], which may make it difficult to keep up with in terms of identification and assessment and potential inclusion on Beall’s list.

It is unclear how journals identify authors to receive these invitations. While the most common invitations received were to submit manuscripts in either endocrine or metabolic diseases, a cursory search of Scopus and Medline did not identify any such publications in the last 3 years for the invitee (DM). It is possible that random trolling of databases, such as PubMed, institutions, and social media, such as Facebook, to identify authors and their associated email addresses is how the invitation lists are generated.

It is rare for journals indexed in Medline or the Directory of Open Access Journals (DOAJ) to send personalized invitations to prospective authors inviting manuscript submission. One exception is an invited commentary or editorial. For example, some journals ask peer reviewers whether the manuscript they are reviewing warrants an editorial and whether the reviewer might be interested in writing it or recommending a recognized expert who could be invited. Many journals have high rejection rates for manuscripts, a phenomenon which is incompatible with inviting this many submissions.

Many of the invitations claimed that their journal and publisher are highly scientific, yet a cursory examination of the journals’ instructions for authors indicated that the majority of them do not mention many of the attributes associated with scientific rigor, such as recommending the use of reporting guidelines. For example, several hundred journals indexed in Medline and Directory of Open Access Journals (DOAJ) endorse the use of CONSORT [10]. Similarly, whether these journals and publishers adhere to publication ethics [11] outlined by the Committee on Publication Ethics (COPE) is doubtful. As part of our ongoing predatory journal research program we are completing a large systematic comparative analysis of these journals.

There are strategies to help minimize receiving these invitations. Many invitations have a journal unsubscribe link. Before deleting the invitation it might be worthwhile spending some time to unsubscribe to the journal. Sometimes a little extra investigation will help identify the publisher and sending a short polite yet firm correspondence asking that your email address be unsubscribed from all the publisher’s journals can be effective. Asking for a receipt of actions taken by the journal and publisher is another option to consider. Another possibility is to ask your institutional information technology group to add the publisher Uniform Resource Identifier link to the current ‘black list’ firewall that many institutions already maintain. Based on the analysis presented here, our institution has blocked the 10 publishers and journals sending the most frequent invitations. Finally, recipients may opt to introduce email filters for the most frequent publishers, thereby blocking many invitations with relative ease. 

For those invitations that do get through to an electronic inbox, there are strategies to consider before responding. Firstly, check whether the journal or publisher is listed on one of Beall’s list of potential predators. While these lists are not perfect, and may not reflect a journal or publisher’s scientific evolution – from having many attributes of a predatory journal to a more legitimate scientifically rigorous journal – they are comprehensive lists, kept up-to-date. Submitting a manuscript to any journal or publisher on the list should be avoided. Secondly, before responding, discuss the invitation with colleagues and mentors. Students should consider consulting with their supervisor and more senior students/post-doctoral fellows within their group.

Researchers earlier in their career might be particularly vulnerable to these invitations. Unfortunately, our current reward system promotes counting of publications rather than other attributes such as completeness of reporting or the ability of others to replicate methods. Promotion and tenure is still geared towards productivity rather than quality. In some settings, a small proportion of salary – merit pay – is based on productivity. Finally, publishing articles in these journals may not be an effective way to let colleagues know about your research. While many of them claim to be open access, they are not indexed in any of the legitimate databases, such as PubMed (many of these journals claim that their publications are indexed in legitimate databases including PubMed; our predatory journal research program is in the process of confirming these claims). As such, they will not be automatically identified by colleagues. They are therefore less likely to be identified for potential inclusion for subsequent systematic reviews and meta-analysis.

Academic institutions might also consider guidance or policy initiatives to safeguard the interests of their research community and trainees. For example, guidance could be for the institutional research community to avoid submitting any manuscript to journals or publishers on Beall’s list. These journals do not appear to have attributes associated with scientific rigor: they are not indexed in reputable databases, and colleagues are very unlikely to be able to identify any such publications. As such, dissemination of the research is likely to be substantially limited. Similarly, these publications are unlikely to positively add to an institution’s reputation. Similar guidance has already been introduced at Savitribai Phule Pune University, India [12].

This study is not without limitations. The analysis of invitations is from a single researcher with an established publication record. To what extent the findings are generalizable is unknown, particularly to researchers earlier in their career who may not have a large publication record. Kozak et al. [13] very recently published a similar paper examining a little more than a year’s invitations (directed to Dr. Kozak, a multi-disciplinary researcher whose research focuses on social science, agriculture, and biology among other topics). Their findings are broadly similar to what we report here. Anecdotally, we are also aware that many other biomedical researchers claim similar types of invitations as those described in this paper. There was no way check the functionality of the unsubscribe links. Finally, this paper did not examine a closely related recent phenomenon, namely, receiving similar invitations to be a speaker at a conference.
